# Turning Polluted Biomass Waste into Sustainable Carbon-Based Catalysts for Hydrogen Production via Water Electrolysis

**DOI:** 10.1021/acs.energyfuels.5c02282

**Published:** 2025-07-23

**Authors:** Jorge Comendador, Javier Llanos, Álvaro Ramírez, Martín Muñoz-Morales, Ester López-Fernández

**Affiliations:** Department of Chemical Engineering. Faculty of Chemical Sciences and Technologies, University of Castilla La Mancha, Campus Universitario s/n, 13071 Ciudad Real, Spain

## Abstract

The development of highly efficient, effective, and low-cost carbon-based catalysts for hydrogen production through water electrolysis represents a significant challenge in sustainable energy conversion. In this work, carbon materials derived from biomass waste, specifically a metal-polluted vegetal species (*)* from a former mining location, were used. Biomass was subjected to hydrothermal carbonization, producing hydrochar. The influence of both thermal and chemical post-treatment was studied in relation to hydrogen production efficiency. The thermal treatment was conducted at 300, 500, and 1000 °C, while the chemical precursors used were KOH and H_3_PO_4_. Additionally, these waste-derived carbon materials were compared with carbon Vulcan XC-72, a common reference material in these processes originated from fossil sources. Several electrochemical techniques were employed to evaluate and identify the most suitable sample for the hydrogen evolution reaction (HER). Additionally, physicochemical characterization analyses were conducted to gain a comprehensive understanding of the morphology, composition, and surface structure of the biomass-derived carbon materials, as well as to establish correlations with their electrochemical behavior toward the HER. The sample that demonstrated the most favorable performance was the one chemically activated with KOH, which exhibited an outstanding Tafel slope (147 mV/dec) and a low overpotential at 10 mA/cm^2^ (−550 mV vs RHE) surpassing even the commercial Vulcan XC-72 sample. Furthermore, the chronoamperometry test showed a very stable performance for this sample. These results demonstrate that plant biomass waste containing metals presents a viable alternative to carbon blacks, commonly used as electrocatalysts for hydrogen production, also providing an efficient and sustainable method to valorize these wastes.

## Graphical abstract

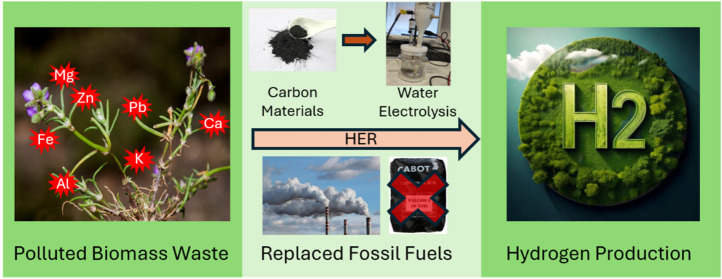

## 1 Introduction

The continuous consumption of energy and persistent environmental pollution of the environment have driven humans to develop clean, renewable, and efficient energy methods and sources.^1^ Renewable energy sources such as solar, wind, and tidal energy can meet energy demands; however, their intermittent nature makes continuous energy generation challenging. Therefore, it is crucial to convert these resources into forms of energy with less external interference.^2^ Hydrogen has gained significant attention from scientists due to its high energy efficiency, lightweight nature, and sustainability. However, its environmental impact and carbon footprint depend on the production pathway. While green hydrogen, produced via water electrolysis by the hydrogen evolution reaction (HER) using renewable energy, is considered a zero-carbon fuel, other types such as gray, blue, and brown hydrogen involve fossil fuels and varying levels of CO_2_ emissions. Therefore, the potential of hydrogen as a clean energy carrier relies on the development and implementation of sustainable production methods.^3^

The electrocatalytic splitting of water is fundamental for the efficient and economical production of hydrogen, which relies on the development of cost-effective and highly active catalysts.^4^ Water electrolysis is a crucial reaction for the storage and conversion of renewable and clean energy. This process involves splitting of water molecules into hydrogen and oxygen by passing an electric current through an aqueous medium. Most hydrogen production processes from water electrolysis use expensive and scarce materials such as Pt, Ir, or Ru, which are considered by the European Union as critical raw materials (CRMs) and increase the cost of the process, making it less economically and environmentally viable.^5^ Furthermore, there is an increase in the consumption of carbon blacks derived also from fossil fuels as a support for catalysts in hydrogen production due to its easy availability and high calorific value. Carbon blacks are highly porous materials, providing a high surface area that facilitates access to active sites, while also exhibiting excellent electrical conductivity properties.^6^ An alternative to the use of commercial carbon blacks, which are typically derived from fossil fuels, is the utilization of residual biomass. When appropriately processed, this biomass can yield materials with similar properties. In the field of hydrogen production, Yang et al. utilized watermelon peels,^7^ while Deng et al. employed animal bones in their investigations, which demonstrated excellent performance in hydrogen production.^8^ In addition, Prabu et al. demonstrated that using palm waste to fabricate hierarchical porous carbon resulted also in highly efficient hydrogen production.^9^

On the other hand, there is currently an environmental issue associated with the accumulation of abandoned or burned waste, which is considered a major cause of environmental pollution, particularly due to greenhouse gas emissions. Therefore, the use of biomass in electrochemical processes is very valuable and significantly enhances the energy recovery of waste.^10,11^ The utilization of biomass derived from mining areas, as employed in this study, addresses two significant current challenges: the production of carbon materials with metals used in hydrogen production (energy-related issue), and the remediation of accumulated biomass, thereby mitigating its associated environmental impact.^12^ While various types of biomasses have been investigated for the hydrogen evolution reaction (HER), the use of heavy metal-contaminated plants from mining environments remains largely unexplored. This approach presents a dual advantage: it enables the development of functional electrocatalysts while simultaneously contributing to environmental remediation. By absorbing heavy metals from contaminated soils, these plants help mitigate pollution and facilitate ecosystem restoration, making their valorization a sustainable strategy that integrates waste management with clean energy production.^13^

In an effort to combine the valorization of carbon residues in electrochemical processes with the valorization of metal-contaminated plant matter, our research group has demonstrated the feasibility of producing a carbon material with properties similar to carbon black from , a plant species that naturally thrives in environments with high metal concentrations. To obtain a material suitable for electrochemical applications, a hydrothermal carbonization (HTC), that is an efficient, low-cost, and environmentally sustainable thermal conversion process,^14^ was carried out to produce hydrochars, followed by chemical or thermal activation steps. The resulting materials exhibited good performance in the electrochemical production of hydrogen peroxide via oxygen reduction^15^ but had not been tested for the hydrogen production reaction, demonstrating their potential to address current energy challenges.

Considering this background, the present study aims to evaluate the feasibility of valorizing the contaminated plant species , subjected to an HTC process, for the production of carbon materials to be used in the electrochemical production of hydrogen. Six samples were used in this study: the first was obtained by filtering and drying the hydrochar; two chemically activated samples, with potassium hydroxide and phosphoric acid, respectively; and three thermally activated samples (at 300, 500, and 1000 °C under N_2_ atmosphere). Physico-chemical and electrochemical characterizations were performed to compare the performance to hydrogen production of the different prepared samples. The results obtained in this study are intended to serve as a basis for future research focused on the valorization of metal-containing plant-based residues, as an alternative to the use of CRMs and fossil fuel-derived carbon products in green hydrogen production.

## 2 Materials and Methods

### 2.1 Reagents

The reagents used in this work are Milli-Q water with a resistivity of 18.2 MΩ·cm. Carbon black Vulcan XC-72 was provided by Fuelcell store. 2-propanol was purchased to Scharlau. Potassium hydroxide pellets 85%, sodium hydroxide pellets 98% and phosphoric acid 85% were supplied by Panreac. Sodium sulfate 99% was provided by Thermo Fisher Scientific. Nafion 5% was brought from Sigma-Aldrich.

### 2.2 Electrode Preparation

#### 2.2.1 Synthesis of Carbon Material

The biomass used in all experiments as raw material was . These wild plants were collected from the abandoned San Quintín mine, located in the mining district of Valle de Alcudia, Ciudad Real, approximately 250 km south of Madrid, Spain. was the only plant capable of growing naturally in the area, retaining high concentrations of heavy metals within its tissues. To ensure reproducibility, all specimens were collected from the same location, where environmental conditions and metal contamination levels are relatively homogeneous. Additionally, the biomass was thoroughly mixed and homogenized before processing to minimize compositional differences between samples.

The samples were subjected to a temperature of 60 °C for 24 h. The next step involved grinding the plant using a RETSCH Ultra Centrifugal Mill ZM 200 (see Figure S1 of Supporting Information (I)).

Once the plant was ground, the HTC process began (II), which is a highly efficient and valuable technology for processing wet solid waste and generating carbon-rich solid materials known as hydrochar. A homogenization of 130 g/L of the sample was prepared in Milli-Q water with constant agitation at 300 rpm for 30 min. This mixture was placed in the autoclave and heated in the oven at 200 °C for 2 h. After reaching ambient temperature, the sample was agitated at 300 rpm for 30 min in 800 mL of water. To separate the hydrochar from the water, a filtration system was employed using a vacuum pump, and the sample was dried in the oven at 100 °C for 8 h (this sample was named as 130/hydro).

Two of the samples were chemically activated, one with 50% KOH (130/600 KOH) and the other with 85% H_3_PO_4_ (130/600 H_3_PO_4_) (III). A weight of 50 g was used for every 100 mL, allowing them to agitate for a few minutes to obtain the chemical solutions. Subsequently, the mixtures were combined in a weight ratio of 1:5 of hydrochar to the weight of the chemical solution and stirred on a heating plate at 300 rpm for 2 h, maintaining a constant temperature of 80 °C. The resulting mixture was then placed in the oven at 100 °C for 24 h.

Thermal activation (IV) was performed on five of the six samples analyzed (all except 130/hydro) by heating the hydrochar to different temperatures under a gas flow of 150 mL N_2_/min in a Forns Hobersal tubular furnace in an inert atmosphere: 300 °C (130/300), 500 °C (130/500), 600 °C (130/600 KOH and 130/600 H_3_PO_4_) and 1000 °C (130/1000). The heating ramp at the start of the program was set to 2 °C/min. The tubular furnace was programmed based on the temperature reached for each sample, maintaining the final temperature for 30 min once it was achieved.

The two samples that were chemically activated were subjected to multiple filtration systems until the used water reached a nearly neutral pH, ensuring the removal of all chemical residues (KOH or H_3_PO_4_) from the activated carbon. The produced carbon material was then utilized for the fabrication of the working electrode and its subsequent use in the electrochemical cell (V).

Therefore, the samples are labeled as 130/X, where 130 refers to the g/L of the initial sample used in the hydrothermal carbonization (HTC) process. X indicates the temperature at which the sample was thermally activated. If the sample was subjected to chemical activation, as in the case of the two samples treated with KOH or H_3_PO_4_, this is explicitly indicated in the label, following the temperature. Additionally, Vulcan XC-72 carbon black (CVulcan) was used as a catalyst for comparative purposes against the biomass catalyst samples.

#### 2.2.2 Fabrication of Working Electrode

To deposit the catalyst layer on the Glassy Carbon (GC), inks were prepared using 2 mg of activated carbon. This carbon material, regardless of sample, was consistently processed following the same procedure. It was mixed with 750 μL of isopropanol and 250 μL of Milli-Q water. Finally, 8 μL of 5 wt % Nafion was added to the sample to control the interaction between the biomass and the solvent, and the mixture was stirred for 30 min. A total of 19 μL of the catalyst ink was deposited on the GC to achieve a catalyst loading of 0.2 mg/cm^2^.^16^

### 2.3 Characterization Techniques

#### 2.3.1 Physical-Chemical Characterization

To analyze the morphological and compositional properties of the biomass, images of the carbon materials surface were captured using high-resolution scanning electron microscopy (HR-SEM) on a ZEISS GeminiSEM 500 microscope. Raman spectrometer Renishaw InVia Reflex with a 532 nm laser basis at room temperature was used to evaluate the graphitic nature and the surface defects. X-ray diffraction (XRD) analysis was performed using a Philips (PANALYTICAL) X’Pert MPD X-ray diffractometer to study the crystallinity of the carbon materials. Diffraction patterns were collected over a 2θ range from 3 to 100°. The identification of crystalline phases in the materials was carried out using the X’Pert HighScore Plus software. Fourier transform infrared spectrum (FT-IR) was used to detect the surface structure of biomass using an infrared spectroscopy equipment, Shimadzu brand, IRAffinity-1S model, with a midband infrared lamp and a standard DTGS detector. X-ray photoelectron spectroscopy (XPS) was used to characterize the elemental composition and chemical state of the sample on the surface analysis equipment model FleXPS.

#### 2.3.2 Electrochemical Characterization

Electrochemical measurements were performed using an ORIGASTAT potentiostat/galvanostat analyzer, model OGS080 from Origalys. The experiments were conducted in a three-electrode electrochemical cell, with a rotating disk electrode (RDE) of glassy carbon of 0.19 cm^2^ used as working electrode, a Hg/HgO electrode as reference electrode, and a graphite rod as counter electrode. Different solutions of 0.1 or 1.0 M of NaOH, Na_2_SO_4_ or KOH or mixed were employed as electrolyte. All experiments were carried out at room temperature.

To analyze H_2_ production, linear sweep voltammetry (LSV) experiments were conducted using a potential ramp from −800 to −1500 mV vs Hg/HgO, with a scan rate of 5 mV/s. Chronoamperometry tests (CA) were performed within a potential range of −1400 mV vs Hg/HgO, lasting for 1 h to observe changes in the hydrogen production over time. Cyclic voltammetry tests (CV) were conducted to calculate the electrochemically active surface area (ECSA), varying the potential from 140 mV to 200 mV vs Hg/HgO, with different scan rates. Electrochemical impedance spectroscopy (EIS) was recorded at a potential of −1400 mV vs Hg/HgO, with frequencies ranging from 1 kHz to 1 mHz, and a wave amplitude of 5 mV.

## 3 Results and Discussion

### 3.1 Physical-Chemical Characterization of Catalysts Derived from Contaminated Biomass

This study presents new images obtained from HR-SEM, as shown in Figure 1, which allows for the analysis of surface characteristics and the microstructures present in all the catalysts. These images, derived from the same materials used in Ramírez et al.,^15^ were newly acquired at different magnifications compared to those reported in the previous study. They provide additional insights into the morphological changes induced by chemical and thermal activation treatments, further complementing the existing characterization. The electrocatalytic performance of these materials in the HER is explored, an aspect not previously investigated. These SEM images, along with a more comprehensive characterization, provide a better understanding of the material’s behavior under reaction conditions and contribute to optimizing its performance for hydrogen production. The pores observed in the carbon sheets consist of thin, interconnected carbon layers, while the irregular arrangements are attributed to variations in the different activation temperatures.^17^ Figure 1a shows the initial morphological properties of the biomass after undergoing hydrothermal carbonization, with its carbon surface remaining practically smooth. Changes in the surface structure begin to appear in the images after thermally activating the biomass. In Figure 1b,c,d, it can be observed that as the activation temperature increases, small bumps and sheet-like structures emerge, which are clearly seen in the 130/1000 sample. This indicates that the surface area is increasing.^8^ Based on the thermogravimetric analysis conducted by,^15^ due to the structural modification and increased specific surface area, it can be stated that at 300 °C, the hemicellulose in the biomass has degraded, followed by the degradation of cellulose when thermal activation reaches 500 °C. For the 130/1000 sample, prior to reaching 1000 °C, hemicellulose, cellulose, and lignin in the structure have already degraded. In the chemically activated samples, Figure 1e,f show more wrinkled textures compared to the rest of the images, indicating the creation of a greater number of active sites on the catalyst surface.^18,19^ During the chemical activation process, there is an increase in the pore diameter and volume, as well as the formation of new pores as a result of the reaction between carbon and KOH or H_3_PO_4_.^19^ In Figure 1e, a mesh-like structure can be observed in 130/600 KOH, which, together with the larger number of pores and its wrinkled texture, enhances the electrochemical activity of this catalyst. The higher porosity observed in the KOH-activated sample can be attributed to the typical mechanisms involved in chemical activation with KOH. At low temperatures, KOH promotes dehydration of carbonaceous compounds, generating water and other intermediates that diffuse into the carbon structure. As the temperature increases, further reactions produce gases such as CO, CO_2_, H_2_, and traces of CH_4_. The release of these gases leads to pore formation within the carbon matrix. Around 500 °C, noncarbon elements volatilize, contributing to structural opening. At approximately 600 °C, KOH reacts completely, forming potassium-containing species such as K_2_CO_3_ and K_2_O, which further enhance pore development. These combined processes explain the significant increase in porosity and surface area compared to the nonactivated samples.^20^ The biomass used in this study contains residual metals as confirmed by energy dispersive X-ray spectroscopy (EDS) analyses (see Tables 1 and S1 in ref 15), consistent with previous work by our research group,^15^ show, respectively, the structural composition of the raw biomass and the surface composition of carbon materials expressed in atomic percentage. These results confirm the presence of metals such as Fe and Zn, which, as reported in the literature, can enhance the electrocatalytic activity toward the HER.^21,22^

**Figure 1:**
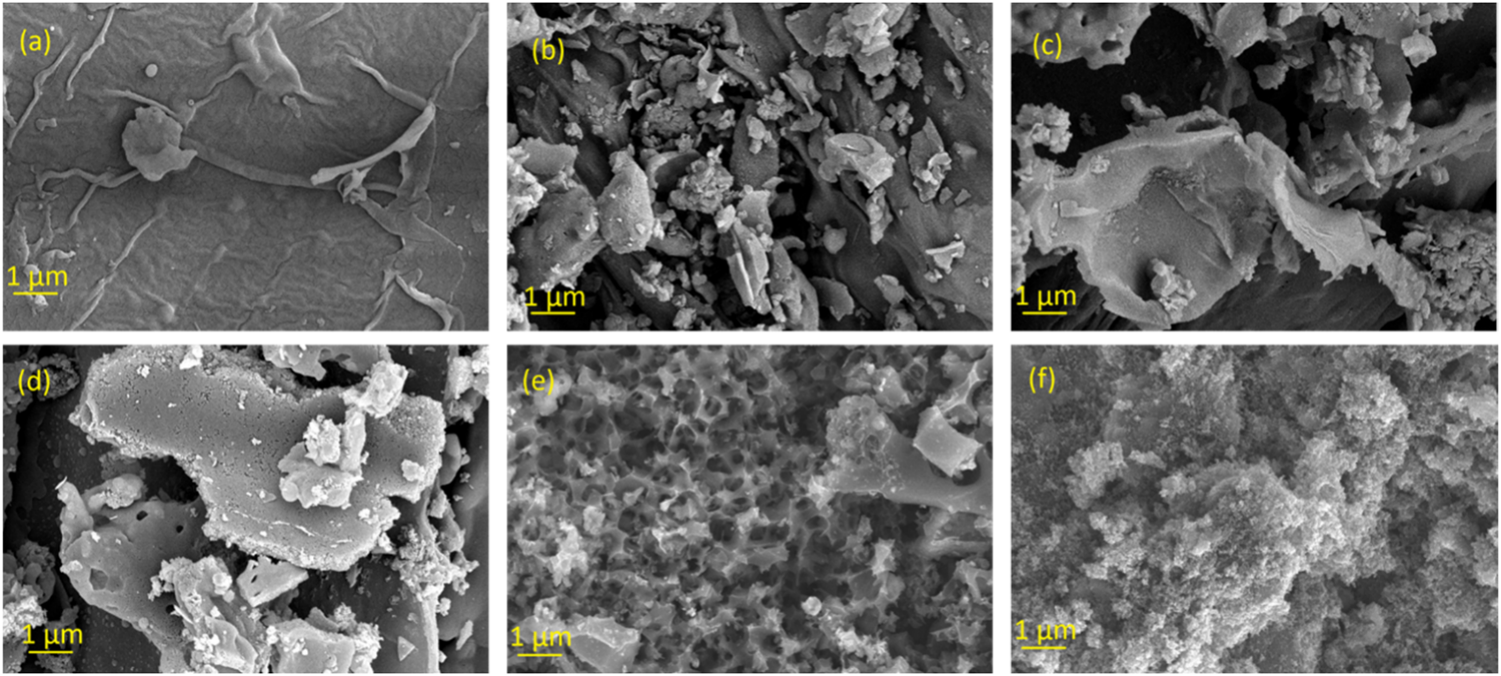
High-resolution SEM images for (a) 130/hydro, (b) 130/300, (c) 130/500, (d) 130/1000, (e) 130/600 KOH, (f) 130/600 H_3_PO_4_.

**Table 1:** EIS Parameters of Different Samples

**sample**	*R*_1_ (Ω)	*R*_2_ (Ω)	*C*_1_ (mF)	*R*_3_ (Ω)	*C*_2_ (mF)
130/hydro	9.33	1749.90	0.003	23811.00	0.11
130/300	15.70	1316.80	0.004	4649.30	0.04
130/500	38.82	203.46	0.006	2247.50	0.35
130/1000	12.34	57.70	0.87		
130/600 KOH	21.67	43.87	1.15		
130/600 H_3_PO_4_	45.33	182.96	1.10		
CVulcan	191.37	2573.30	0.62		

Next, Figure 2 shows XRD patterns, Raman spectra and FT-IR spectra of all materials synthesized.

**Figure 2:**
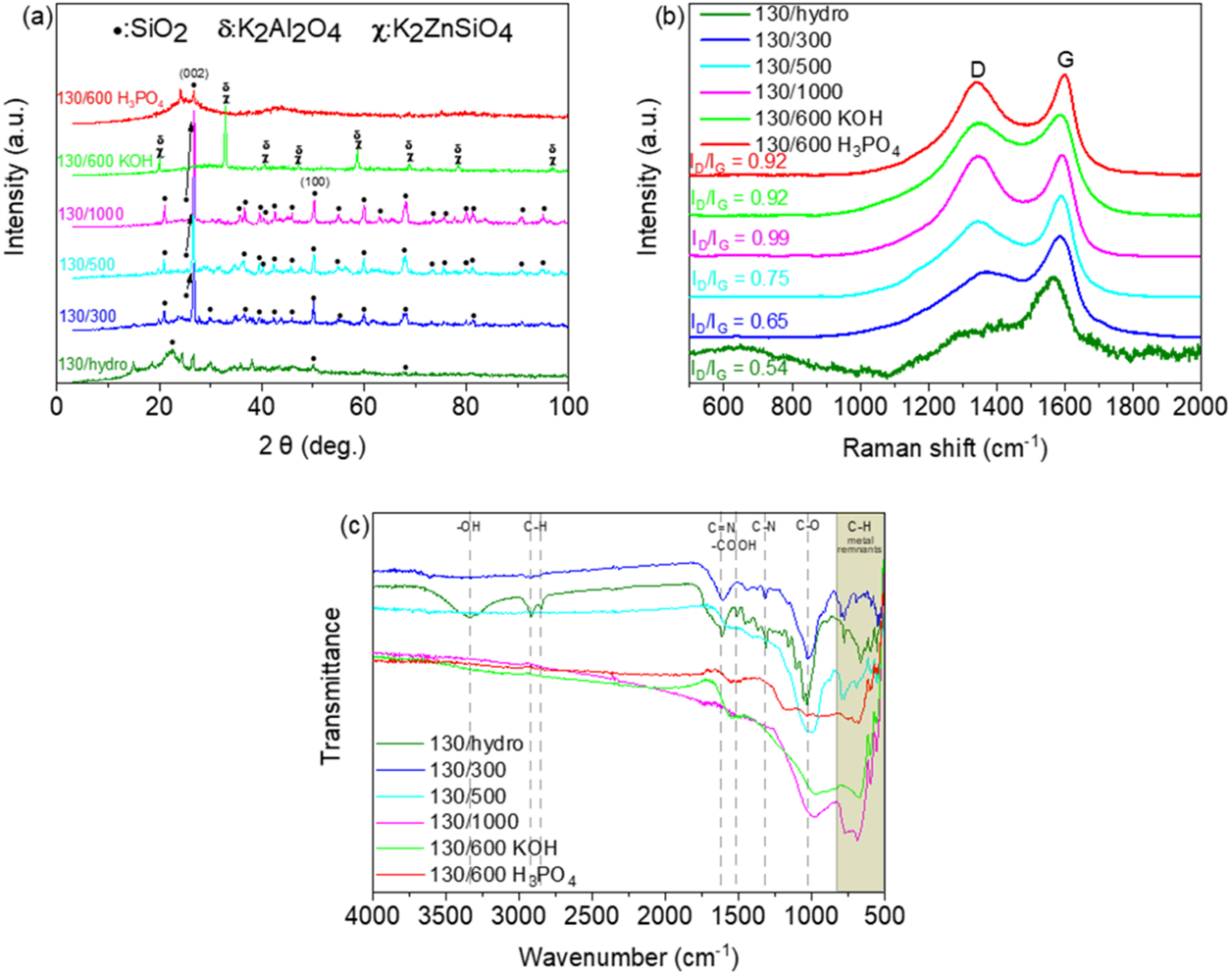
(a) XRD patterns, (b) RAMAN spectra and (c) FT-IR spectra of contaminated biomass catalysts.

XRD analysis is an important technique for identifying the composition and crystallinity of the developed materials. The XRD pattern (Figure 2a) revealed that the thermally activated materials exhibit similar crystal structures and distinctive characteristics, whereas the chemically activated samples follow a completely different pattern. The effect of temperature on the crystallinity of the samples is evident, as increasing the activation temperature from 300 to 1000 °C results in the spectral peaks becoming stronger, more defined, and sharper. This improvement in peak clarity is attributed to crystal formation within the catalysts.^23^ The crystalline phases in the materials were identified using the ICDD database, which allowed for the accurate assignment of the observed peaks in the X-ray diffraction patterns to their corresponding phases.

This XRD pattern shows the peaks of the carbon structure (002) and (100)^24^ with broader peaks around 26.7 and 50.2°, respectively, with the former being very prominent in the 130/500 and 130/1000 samples. The presence of the (100) peak suggests the formation of condensed graphite. These characteristic planes (002) and (100) in the samples exhibit interlayer spacing of approximately 0.33 and 0.18 nm, respectively. In the chemically activated samples with KOH, the (002) and (100) peaks disappear due to the crystallization process generated during chemical activation, indicating that KOH destroyed the graphitic crystalline domains in the carbon structure, enhancing the accessibility and dispersion of electrolyte ions and contributing to better electrochemical performance.^25^ In contrast, the (100) peak also disappears in 130/600 H_3_PO_4_, resulting in more defects in the amorphous carbon structure.^26^ A broad peak appears around 43° in this sample,^27^ which is likely related to the transformation of the organic crystalline compound into microcrystalline carbon with a fine-grain graphitization structure, so this is beneficial for achieving efficient electrochemical performance.^28^

In Figure 2a, the most significant peaks for each sample are represented due to their relevance in the HER. In the catalysts that were not chemically activated, quartz (SiO_2_) predominates, which is a mineral composed of silicon and oxygen atoms, so it is beneficial for HER as a structural support. In 130/hydro sample the first traces of quartz are observed at the SiO_2_ peaks at 24.4, 50.2, and 68.0° (PDF reference card: 01-078-1252). The samples 130/300, 130/500, and 130/1000 exhibit a similar pattern regarding the number and shape of the peaks. The most prominent peak corresponds to the carbon structure (002) at 26.7°, clearly standing out in the graph, along with the carbon peak (100) at 50.2° and a peak around 68° that appears in all three catalysts, with all peak sizes increasing as the thermal activation temperature rises. In the 130/300 sample, the presence of SiO_2_ begins to be noticeable, observed at the peaks 15.0, 26.7, 29.8, 36.7, 39.5, 42.4, 45.8, 50.1, 55.2, 60.0, 68.3, and 81.3° (01-085-0794). In the 130/500 sample, SiO_2_ is observed at peaks 20.8, 26.7, 36.6, 39.4, 40.2, 42.4, 45.8, 50.1, 54.8, 59.9, 68.1, 74.4, 75.5, 79.9°, and is also reflected in the more distant peaks at 90.8 and 94.9° (01-085-1054). In the 130/1000 sample, the presence of SiO_2_ increases, appearing at the same peaks as in 130/500, along with a peak at 31.9° and a new peak at 63.1°, which is only recorded in this catalyst (01-078-1254). For the catalysts that have been chemically activated, the number of peaks is significantly reduced, especially in the case of 130/600 H_3_PO_4_, where only SiO_2_ data is recorded at peak (002) at 26.1°, along with two very small peaks at 68.8 and 75.2° (01-085-0335). In 130/600 KOH, the effect of KOH in the chemical activation is highlighted by the presence of K in the sample, where data for K_2_ZnSiO_4_ and K_2_Al_2_O_4_, which can enhance conductivity and stabilize catalytic intermediate states, accelerating HER, are recorded at peaks 20.0°, and a very prominent peak at 32.9, 38.7, 47.7, 58.7, 68.9, 78.4, and 96.8° (00-039-0268 and 00-045-0849) (all XRD peaks are represented in Figure S2).

The degree of graphitization of the carbon materials was measured using Raman spectroscopy (Figure 2b), which shows two distinct peaks at 1349 and 1591 cm^–1^, corresponding to the D band and the G band, respectively. The D band refers to the amorphous carbon or structural defects, which can increase the active area; while the G band corresponds to the graphite structure, reflecting the degree of graphitization and contributing to higher electrical conductivity. The ratio of the D band to the G band intensity (*I*_D_/*I*_G_) was utilized to assess the structural defects in the carbon-based materials.^9^ After calculating the intensity ratio of D and G bands (*I*_D_/*I*_G_), the following results were obtained: 0.54 for 130/hydro, 0.65 for 130/300, 0.75 for 130/500, 0.99 for 130/1000, 0.92 for 130/600 KOH and 0.92 for 130/600 H_3_PO_4_. This shows that as the activation calcination temperature increased, the active area became higher.^7^ The absence of additional peaks or bands in the Raman spectrum demonstrates that there are no other secondary impurities in the catalysts, as the carbon materials synthesized present a highly porous structure when chemically activated with KOH.^26^

In order to more accurately represent the degree of structural defects in the biomass, the ratio A_D1_/A_G_ was also calculated based on the structural parameters obtained from the Raman data analysis, employing a 5-band model, as shown in Figure S3. In this ratio, a higher value reflects a greater degree of structural defect.^29^ The samples 130/hydro, 130/300, and 130/500 indicate an increase in the ratio A_D1_/A_G_ as the activation temperature rises, with results of 0.86, 1.08, and 1.15, respectively. In the samples 130/1000, 130/600 KOH, and 130/600 H_3_PO_4_, the degree of structural defects in the biomass has significantly increased, with A_D1_/A_G_ values of 1.49, 1.63, and 1.78. Contrary to their A_D1_/A_G_ratios, the chemically activated catalysts show a higher proportion than 130/1000. These results indicate that the three catalysts present a high level of defects in their carbonaceous structures,^30^ creating additional active sites that enhance the HER.^31^ Although subsequent results will demonstrate that the electrocatalytic activity is higher in the biomass activated with KOH than with H_3_PO_4_, the latter shows a greater A_D1_/A_G_, likely caused by the addition of oxygenated or phosphorated functional groups during chemical activation, contributing to a higher degree of structural disorder.

In Figure 2c, the chemical changes in the structure of the six catalysts were studied using FT-IR spectra. The presence of a wide number of bands indicates that hydroxyl groups have not been removed during the activation treatments.^16^ In 130/hydro, three bands of −OH groups are clearly observed at approximately 3400 cm^–1^ and C–H groups at 2900 and 2850 cm^–1^, but these disappear in all the catalysts that have undergone activation processes.^32,33^ Starting from a lower wavelength, four peaks can be observed in all samples at approximately 550, 600, 700, and 800 cm^–1^indicating the presence of aromatic compounds (C–H) in their structures.^34,35^ With the help of XRD analyses, it can be identified in FT-IR spectra that remnants of metals appear at these peaks, with a distinctive peak in the catalysts that have not been chemically activated at approximately 800 cm^–1^ and another peak in all samples at 700 cm^–1^. Both C–H peaks may be influenced by the presence of these metals, especially of Si in the various forms it has been recognized, mostly in SiO_2_, and Al in the form of AlO, as well as a combination of both metals (Al–O–Si).^36^ Additionally, Fe is located at the peaks of 550 and 600 cm^–1^, likely in the form of FeO,^37^ with the most prominent peaks found in the 130/1000 sample, which is the only one in which this compound will be represented in the XPS spectrum. To a greater or lesser extent, an absorption peak appears at approximately 1000 cm^–1^, caused by asymmetric stretching vibrations corresponding to the C–O group.^17^ Around 1550 cm^–1^, small vibrations corresponding to −COOH groups are found only in the hydrochar and in the chemically activated samples.^38^ A small peak appears only at around 1300 cm^–1^ in 130/hydro and 130/300 corresponding to C–N stretching modes. The absorption bands around 1650 cm^–1^ and little peak at 2350 cm^–1^are assigned to the vibrational modes of C = N and C≡N bonds.^39^

Next, XPS characterization was also performed and represented in Figure 3. Based on the results from the XPS analysis (see general spectra in Figure S4), a series of peaks corresponding to the functional groups found in each of the samples was obtained. The scans recorded the core levels of C 1s, O 1s, N 1s, Zn 2p, Si 2p, Zn 3p, Al 2p, Ca 2p, Fe 2p, and P 2p.

**Figure 3:**
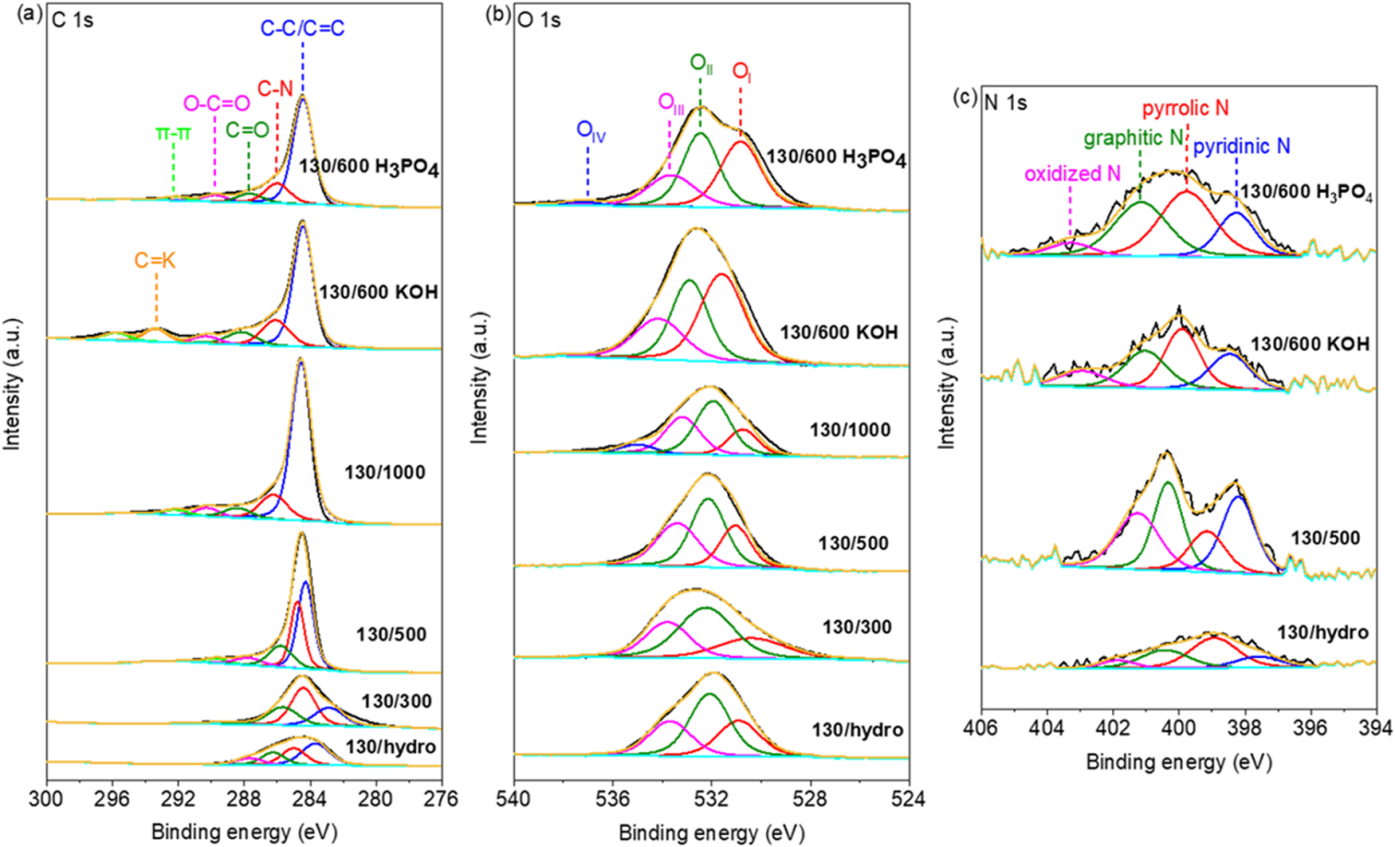
Core level high-resolution C 1s (a), O 1s (b) and N 1s (c) XPS spectra of contaminated biomass catalysts.

In Figure 3a, the C 1s spectra of all catalysts exhibits four main components, centered approximately at 284.4, 286.1, 288.2, and 290.3 eV, corresponding to C–C, C–N, C = O, and O–C = O, respectively. Additionally, a further peak around 292.2 eV is observed, attributed to π–π transitions in the catalysts activated at temperatures above 500 °C.^40,41^ 130/600 KOH sample also displays a contribution at 293.6 eV, associated with the C–K bond, formed during the KOH activation process, which is supported by the exclusive presence of the K 2p signal in this catalyst. For 130/1000, 130/600 KOH and 130/600 H_3_PO_4_, a significant increase in the C–C/C = C contribution was reported, indicating the presence of both sp3 and sp2 hybridized carbon species.^1,7^ In particular, the presence of sp2 graphite carbon contributes to high electrical conductivity, structural stability, and a suitable surface to optimize the HER.^21,42,43^ The O 1s spectrum is represented in all catalysts, as shown in Figure 3b, and reveals the presence of three distinct oxygen species. These are attributed to lattice oxygen (O_I_), OH^–^ and defects (O_II_) and electrophilic oxygen species (O_III_), located at binding energies of approximately 534.2, 532.9, and 531.6 eV, respectively.^41^ In addition, a minor fourth component (O_IV_) was detected in the 130/1000 and 130/600 H_3_PO_4_ samples, which can be attributed to the presence of absorbed water molecules, located at 535.0 and 537.2 eV, respectively.^44^ For chemically activated catalysts, particularly 130/600 KOH, a significant increase in lattice oxygen was observed, which is correlated with enhanced HER activity.^45^ The N 1s spectrum (Figure 3c) was resolved into four distinct nitrogen species: pyridinic N (398.5 eV), pyrrolic N (399.9 eV), graphitic N (401.0 eV), and oxidized N (402.9 eV). The proportion of graphitic N remained relatively constant. Additionally, a slight increase in pyridinic N content and a high retention were observed in the chemically activated samples, which may be associated with a greater number of active sites and enhanced electron transfer, thereby improving the performance of HER.^46–48^

Additionally, Figure S5a presents the spectra of Si 2p, Zn 3p, and Al 2p, due to the proximity in binding energy of the three compounds. The Si 2p peak is located at 102.9 eV in the samples 130/300, 130/500, 130/1000, and 130/600 KOH, corroborating the analysis conducted in Figure 2, where SiO_2_ was the compound with the highest representation in the XRD patterns. Due to the high activation temperatures to which the 130/1000 sample was subjected, Zn 3p and Al 2p peaks are not discernible in the study of this catalyst. The Zn 3p peak is slightly shifted to the left of the central levels of Si 2p, at 89.5 eV.^49^ The Al 2p spectrum disappears in the 130/600 KOH sample, as observed in the XRD study, and is found in the other two samples at 74.6 eV, attributed to Al–OH.^50,51^ Figure S5b illustrates the peaks of the Zn 2p spectrum that appear. These peaks are found at 1021.9 and 1045.0 eV, corresponding to Zn 2p_3/2_ and Zn 2p_1/2_, respectively.^52,53^ The Fe 2p spectrum is clearly observed in the 130/600 H_3_PO_4_ sample, displaying two peaks in Figure S5c separated by 13.6 eV. The first peak is located at 711.2 eV, referenced as the Fe 2p_3/2_ peak, which is the strongest emission peak, while the second peak is found at 724.8 eV, designated as Fe 2p_1/2_, which represents the weaker emission peak situated at a higher binding energy.^54^ The spectrum of Ca 2p appears in the samples of 130/500 and 130/600 KOH, with the most prominent peaks in the former (Figure S5d). The spectra display the Ca 2p_3/2_ and Ca 2p_1/2_ components of the 2p level, with an intensity ratio of 2:1 between them, separated by 3.5 eV due to spin–orbit coupling. The Ca 2p_3/2_ component is located at 347.5 eV, while the Ca 2p_1/2_component appears at 351 eV.^7^ The P 2p spectrum is observed only in the 130/600 H_3_PO_4_ sample due to the P incorporated during the chemical activation with H_3_PO_4_. As shown in Figure S5e, this peak is located at 132.8 eV, attributed to P–C.^7^

### 3.2 Electrochemical Characterization of Contaminated Biomass Catalysts for HER

The electrocatalytic activities for HER were evaluated to determine which of the catalysts derived from carbon samples synthesized from through various activation processes exhibits the highest efficiency in hydrogen production, using electrochemical techniques, including LSV, ECSA determination, EIS, and CA. Additionally, CVulcan was measured for comparative efficiency evaluation. All analysis were carried out under an electrolyte solution of 1 M Na_2_SO_4_ and 0.1 M NaOH at room temperature. This is because this solution provided the best results compared to other electrolytes tested: 1 M KOH, 0.1 M KOH, 1 M NaOH, 0.1 M NaOH, 0.1 M Na_2_SO_4_, and 0.1 M KOH + 1 M Na_2_SO_4_ (see LSV and CA in Figure S6). First, LSV analysis is represented in Figure 4.

**Figure 4:**
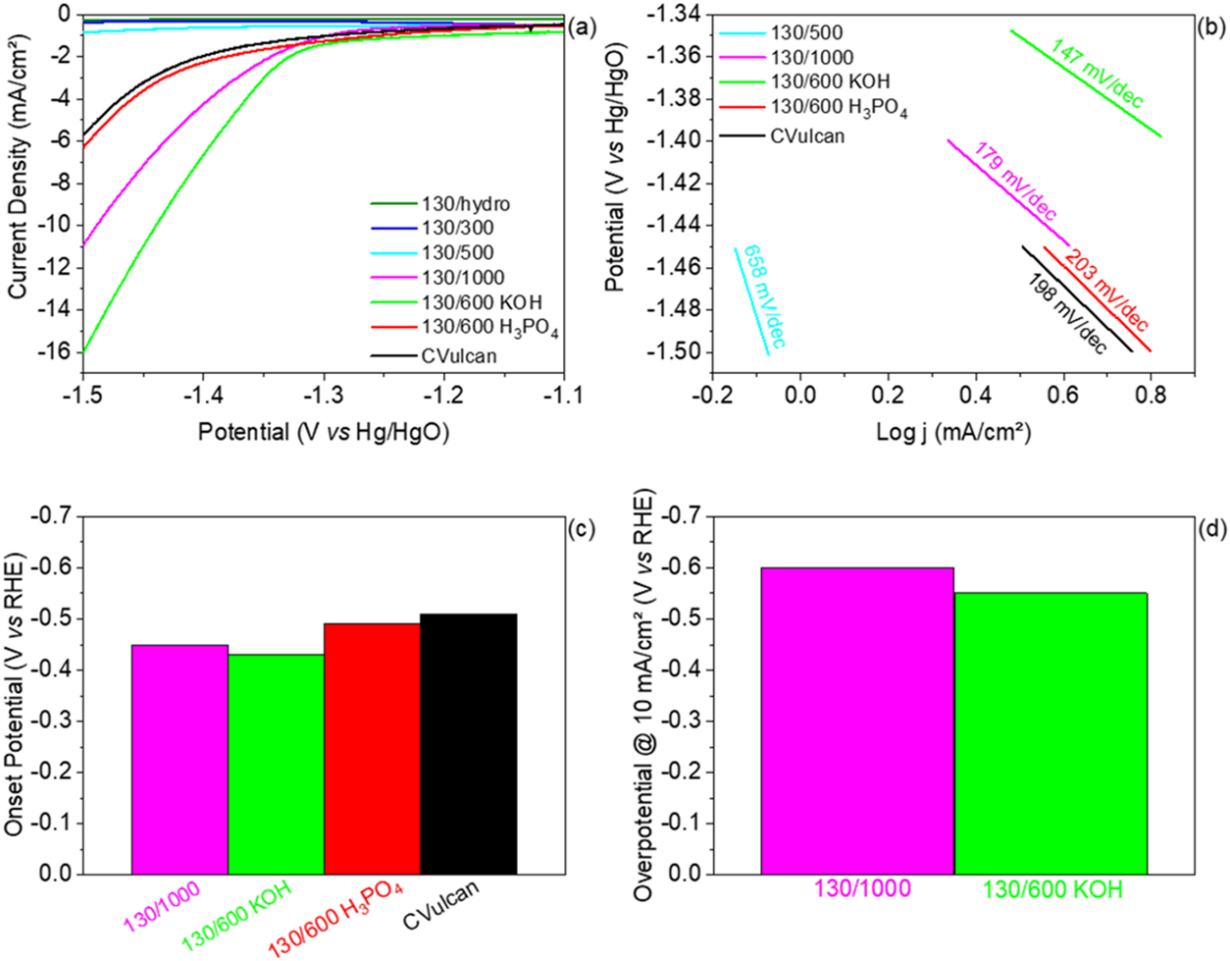
(a) LSV curves for HER using a potential ramp from −800 to −1500 mV vs Hg/HgO, using a scan rate of 5 mV/s of treated biomass catalysts and CVulcan; (b) Tafel curves for 130/500, 130/1000, 130/600 KOH, 130/600 H_3_PO_4_ and CVulcan; (c) onset potential for 130/1000, 130/600 KOH, 130/600 H_3_PO_4_ and CVulcan at −2 mA/cm^2^ and (d) overpotential to generate a current density of 10 mA/cm^2^ for 130/1000 and 130/600 KOH.

Raw LSV tests, correlated to the electrocatalytic performance of the samples, are shown in Figure 4a. The current density is directly proportional to the rate of hydrogen evolution, as it represents the number of electrons transferred per unit area during the reaction. A higher absolute current density at a given applied potential indicates an increased electrochemical activity, leading to a greater hydrogen production rate. The 130/600 KOH sample exhibits a steeper slope in LSV curve compared to other materials, followed by 130/1000 and 130/600 H_3_PO_4_, surpassing the results obtained with CVulcan. This demonstrates the potential of these biomass-derived materials to replace carbon Vulcan, thereby reducing reliance on fossil-derived materials in hydrogen production while simultaneously promoting waste valorization. On the other hand, samples 130/hydro and 130/300 showed insignificant electrocatalytic activities, discarding them, therefore, for efficient hydrogen production. To further analyze the results obtained from the LSV for HER, Tafel slopes were calculated, as graphically shown in Figure 4b. Generally, a lower Tafel slope value indicates a greater increase in current density with the same overpotential, providing a more efficient HER process.^4,31^ The Tafel slope value for 130/600 KOH sample was 147 mV/dec lower than the other samples studied; exhibiting the highest catalytic performance for HER (significantly superior to the CVulcan sample), indicating a faster electron transfer rate and higher hydrogen generation compared to all the catalysts studied.^55^ There are generally two mechanisms for HER kinetics in alkaline media: (i) Volmer-Heyrovsky and (ii) Volmer–Tafel. The Tafel value obtained for the 130/600 KOH electrocatalyst (147 mV/dec) indicates that the initial Volmer step involves hydrogen adsorption on the catalyst surface, followed by either electrochemical desorption or hydrogen recombination. Therefore, the HER performance predominantly follows the Volmer-Heyrovsky mechanism.^21^ Tafel slope value obtained for 130/600 KOH sample was lower than others values reported in literature for biomass carbon catalysts used in HER, such as for*Acorus Calamus*plant (420 mV/dec),^56^ biomass waste derived from the fruit shells of*Tamarindus indica*(204 mV/dec),^32^ and carbon materials derived from waste grapefruit peel (198 mV/dec),^57^ indicating a better electrochemical activity to H_2_ evolution of the material tested in the present work. Figure 4c shows the onset potential values for hydrogen production, which refer to the potential at which the catalytic current begins to appear, defined using a threshold of −2 mA/cm^2^to allow for comparison among the catalysts.^58^ 130/1000 and 130/600 KOH samples exhibited the lowest onset potential values (−450 and −430 mV vs RHE, respectively), indicating that a lower voltage was required to initiate the HER, making the catalysts more energy-efficient.^59^ These potentials were converted to the reversible hydrogen potential (RHE) using the eq 1:^60^

1 $$E_{\mathrm{RHE}}=E_{\mathrm{Hg}/\mathrm{HgO}}+0.059\cdot \mathrm{pH}+E_{\mathrm{Hg}/\mathrm{HgO}}^{{}^{\circ}}$$

where *E*_Hg/HgO_^°^ = 98 mV and electrolyte pH = 13.42. For 130/hydro, 130/300, and 130/500 samples, onset potential is not clearly observed due to their low electrocatalytic activity for the HER. Based on the obtained values and the physical-chemical characterization performed, it was found that increasing the activation temperature and chemical activation lead to enhanced electrical conductivity, a larger active surface area, and a more ordered structure, factors that contribute to improved electrocatalytic activity in the hydrogen evolution reaction.^3^

In addition, overpotential values for HER were calculated. Overpotential refers to the extra voltage required beyond the theoretical equilibrium potential to drive the reaction at a given current density. A lower overpotential value indicates that the catalyst requires less energy to produce an amount of hydrogen, which is directly related to higher catalytic activity. This is because a catalyst with a lower overpotential can facilitate the reaction more efficiently, resulting in improved hydrogen production rates. Several studies^61,62^ have shown that minimizing overpotential is a key factor in enhancing electrocatalytic performance for hydrogen production. According to the Nernst equation, the potential value of the reversible hydrogen electrode (*E*_RHE_) corresponds to the overpotential for the HER. As shown in Figure 4d, 130/600 KOH and 130/1000 samples required overpotentials of −550 and −600 mV vs RHE, respectively, to generate a current density of 10 mA/cm^2^. This value for 130/600 KOH sample overcomes results of other materials reported in the literature as holey and wrinkled flash graphene (−613 mV vsRHE)^63^ or eggshell membrane with NiO nanoparticles encapsulated (−565 mVvsRHE),^64^ and are similar to others carbon-based catalysts derived from graphene synthesized with nitrogen (−490 mV vs RHE) and phosphorus (−553 mV vs RHE).^65^ To compare with CVulcan sample, overpotentials were obtained at a current density of 5 mA/cm^2^, as CVulcan sample does not reach a current density of 10 mA/cm^2^ under the tested conditions (Figure S7). The results align with the Tafel slopes, with the overpotential of 130/600 H_3_PO_4_ (−590 mV vs RHE) being similar to that of CVulcan (−600 mV vs RHE). Additionally, 130/600 KOH (−490 mV vs RHE) and 130/1000 (−520 mV vs RHE) clearly outperform this carbon black, particularly in the chemically activated sample.

As expected, the electrocatalytic activity of the materials presented here does not match the best results previously reported in the literature for catalysts based on platinum-group metals.^66,67^ Nevertheless, it is worth noting that the electrocatalytic performance obtained in the present work ranks among the highest reported for waste-derived carbon materials that do not rely on the use of critical raw materials (CRMs) to enhance the HER.

To determine the ECSA, CV analysis was performed at different scan rates (5, 10, 20, 50, and 100 mV/s) using potential range from 140 to 200 mV vs Hg/HgO. This potential range was chosen as it is free from significant faradaic processes, allowing for an accurate estimation of the double-layer capacitance (*C*_dl_). Since ECSA is proportional to double-layer capacitance, these CV measurements enable its calculation (see Figure 5), providing insight into the electrochemically active surface area available. The ECSA represents the electrochemically active surface area of a material, meaning the actual area accessible for electrochemical reactions compared to the geometric area of the electrode.

**Figure 5:**
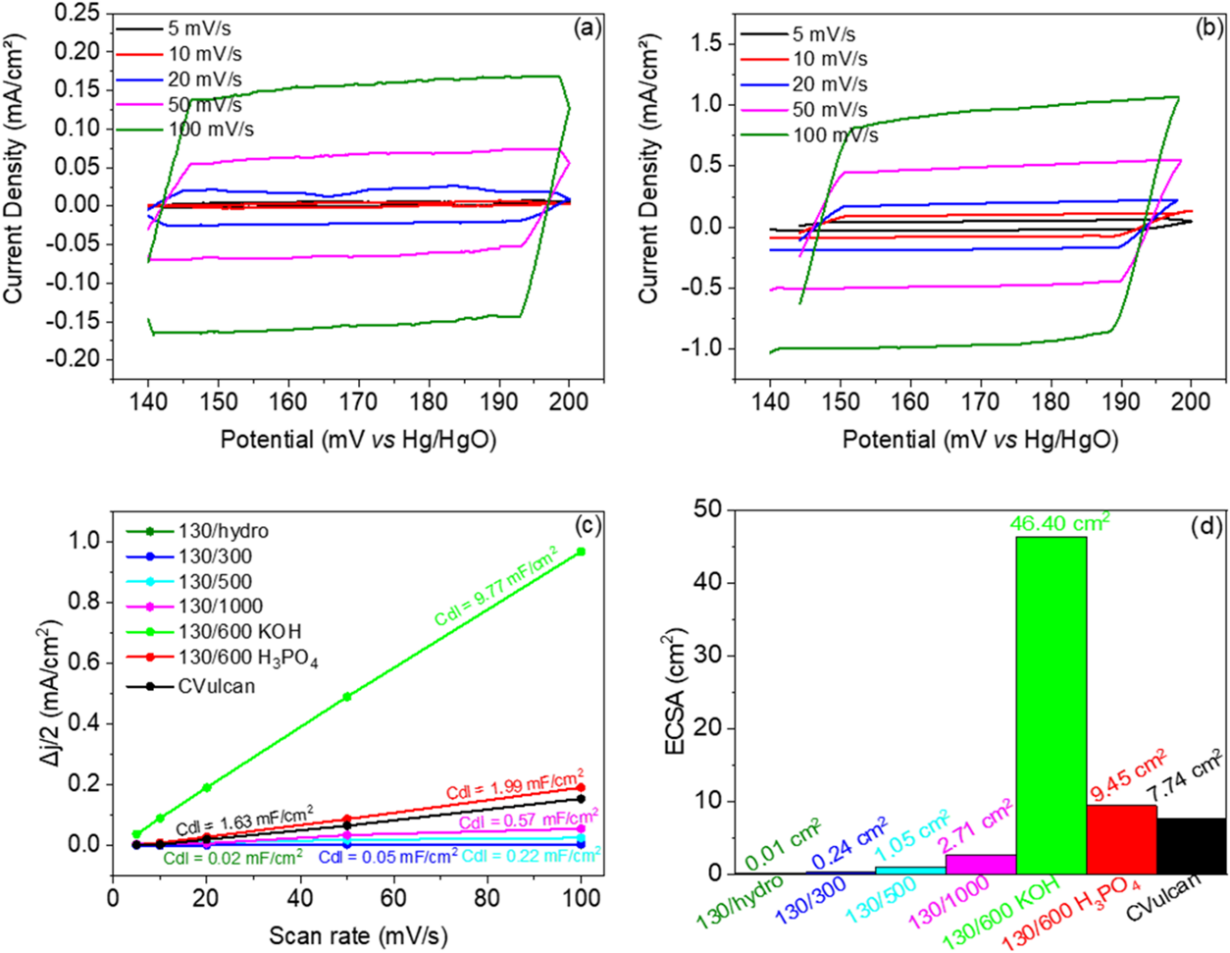
CV plots recorded at scan rates ranging from 5 to 100 mV/s with a 0.1 M NaOH + 1 M Na_2_SO_4_ solution for (a) CVulcan and (b) 130/600 KOH. (c) Current density vs scan rate determined from CVs of the contaminated biomass catalysts and CVulcan with double layer capacitance *C*_dl_ in each electrode. (d) ECSA values in cm^2^.

There are several methods to determine ECSA value for a material. One of them is through the double-layer capacitance, that is commonly used to evaluate carbon materials. The double layer capacitance *C*_dl_ (mF) was determined from the slope of the linear regression of current density values vs scan rate, measured at 170 mV vs Hg/HgO. Based on the *C*_dl_ and *C*_s_ values, the ECSA can be estimated using the eq 2:^68^

2 $$\mathrm{ECSA}=\frac{C_{\mathrm{dl}}}{C_{s}}$$

where *C*_s_ (mF/cm^2^) is the specific electrochemical double-layer capacitance. Specific capacitances have been measured for a variety of electrodes in alkaline solutions and typical values reported range between 0.022–0.130 mF/cm^2^ in NaOH and KOH solutions.^69^ In this case, the chosen value was determined in 0.04 mF/cm^2^, based on the data reported in the existing literature.^29,69^

As shown in the graphs in Figure 5a,b, the current values increase as the scan rate increases. This trend is more clearly observed with higher thermal activation temperatures and is especially pronounced in chemically activated samples (see Figure S8 to see the rest of the CVs of the other samples), particularly in 130/600 KOH (Figure 5b). As illustrated in Figure 5b, this sample reaches a significantly high current density, surpassing the performance of CVulcan (see Figure 5a). These results can be attributed to the higher double-layer capacitance, which corresponds to a larger ECSA in the material. This increased ECSA provides more active sites for the HER, thereby improving the catalytic performance.^70^

As shown in Figure 5c, 130/600 KOH exhibits a high value (9.77 mF/cm^2^), which is consistent with its exceptionally large BET surface area (1097 m^2^/g) (values reported in Table S2 of Ramírez et al.^15^). This correlation highlights the effectiveness of KOH activation in significantly enhancing the material porosity and specific surface area, thus increasing the electrochemical double-layer capacitance. In addition to this sample, 130/600 H_3_PO_4_ (1.99 mF/cm^2^) is the only other sample that surpasses the results of CVulcan (1.63 mF/cm^2^), and large BET surface area (704 m^2^/g). ECSA values, obtained from eq 2 and represented in Figure 5d, validate this trend. 130/600 KOH sample achieves an outstanding ECSA of 46.40 cm^2^, followed by 130/600 H_3_PO_4_ with 9.45 cm^2^, both of which outperform CVulcan (7.74 cm^2^). The superior ECSA values of the chemically activated samples can be attributed to their significantly higher BET surface areas, which provide greater accessibility for electrolyte interaction, facilitate the exposure of active sites, and enhance catalytic activity during HER.^71^ In contrast, the samples without chemical activation display much lower BET surface areas, which are reflected in their lower ECSA values. Particularly, the 130/1000 sample exhibits a low ECSA value of 5.64 cm^2^, despite having a relatively high BET surface area (283 m^2^/g). This discrepancy likely arises from the heterogeneity issues caused by excessive amorphous carbon defects at elevated synthesis temperatures, which compromise the uniformity of active site distribution and the material’s electrochemical performance.^72^

The EIS method was conducted to examine the catalyst’s interfacial behavior and transport characteristics.^73^ Nyquist plot is presented in Figure 6a,b (at different scales due to the visualization range). From the analysis of the Nyquist plot, it was observed that the 130/600 KOH sample exhibited a smaller semicircle compared to the other samples. A smaller semicircle indicates a more efficient charge transfer process, which reduces energy losses and enhances the overall electrocatalytic performance, ultimately leading to higher HER activity.^17^ An equivalent circuit was simulated for better interpretation of the EIS data, as shown in inset of Figure 6a, which consists of R_1_ representing the electrolyte resistance (*R*_s_), and two additional RC (resistor-capacitor) elements.^73,74^ The capacitive components reflect the double-layer capacitance formed at the electrode surface. The resistances R_2_ and R_3_ can be related to the charge-transfer process and the surface porosity, respectively.^73^ The values of all resistance and capacitance values obtained from fitting the equivalent circuit to the EIS data are summarized in Table 1. This approach allows for a more precise understanding of the electrochemical behavior of the catalysts, providing insights into the resistance characteristics and capacitance values, which are essential for evaluating the material overall efficiency in the HER.

**Figure 6:**
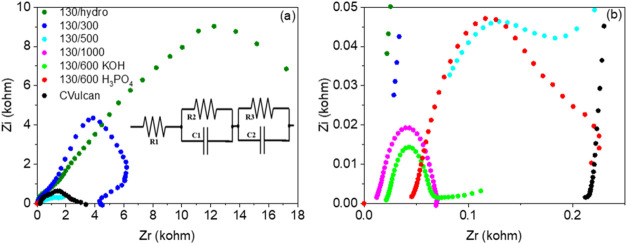
(a) Nyquist plots of the contaminated biomass catalysts and CVulcan with the equivalent circuit and (b) the same figure enlarged for better visibility.

Among all samples, the lowest resistance values (R1 and R2) were recorded for the 130/600 KOH and 130/1000 materials, indicating enhanced ionic conductivity and more efficient charge transfer at the electrode–electrolyte interface.^75^ Importantly, a third resistance element (R3) was required in the equivalent circuit only for the samples treated at 500 °C or below (i.e., 130/hydro, 130/300, and 130/500), suggesting the presence of an additional process hindering charge flow. This resistance can be associated with surface-related limitations due to lower porosity, which restricts effective electrochemical interaction throughout the material. This interpretation is supported by SEM images (see Figure 1), where these low-temperature samples exhibit significantly less-developed porous structures compared to the more activated and highly porous surfaces seen in the 130/600 KOH and 130/1000 samples. This enhanced charge transfer efficiency plays a crucial role in improving the overall electrocatalytic performance, contributing to higher HER activity and reducing energy losses during the process. Note that these values of resistances are lower than those of other HER catalysts, such as Mo-doped Ni_3_S_4_on carbonized wood (74.8 Ω)^75^ and nitrogen self-doped bean sprout (146 Ω).^76^

Finally, the degradation of the electrode or long-term stability is a crucial metric for materials used in HER. Catalyst stability is one of the primary criteria for real-time applicability of these materials.^77^ To study the stability of materials, two common methods can be employed, repetitive cyclic voltammetries or durability experiments (chronoamperometry/chronopotenciometry). For this reason, chronoamperometry was employed to assess the durability of the catalysts in the same aqueous solution of 1 M Na_2_SO_4_ + 0.1 M NaOH (see stability of 130/600 KOH sample in the other solutions in Figure S6b), maintaining a constant potential of −1400 mV vs Hg/HgO for 1 h. The results from CA tests are shown in Figure 7a, where it can be observed that 130/600 KOH continues to exhibit an outstanding catalytic current density compared to the other catalysts, along with good performance in the 130/1000 sample. From the values obtained in CA, the degradation percentage (%) of the samples was determined. Figure 7b shows that the experiments with the lowest degradation percentages are 130/1000, 130/500, and 130/600 KOH. These findings are consistent with previous sections, indicating that these samples are the most suitable for hydrogen production. The high levels of degradation observed may be attributed to potential delamination of the material on the Glassy Carbon. Figure 7c shows a CA test carried out over 24 h for 130/600 KOH, selected due to its superior electrocatalytic performance for HER. This experiment aimed to further evaluate its durability under continuous operation, simulating a more realistic operating scenario. Minimal degradation was observed, confirming its excellent stability.

**Figure 7:**
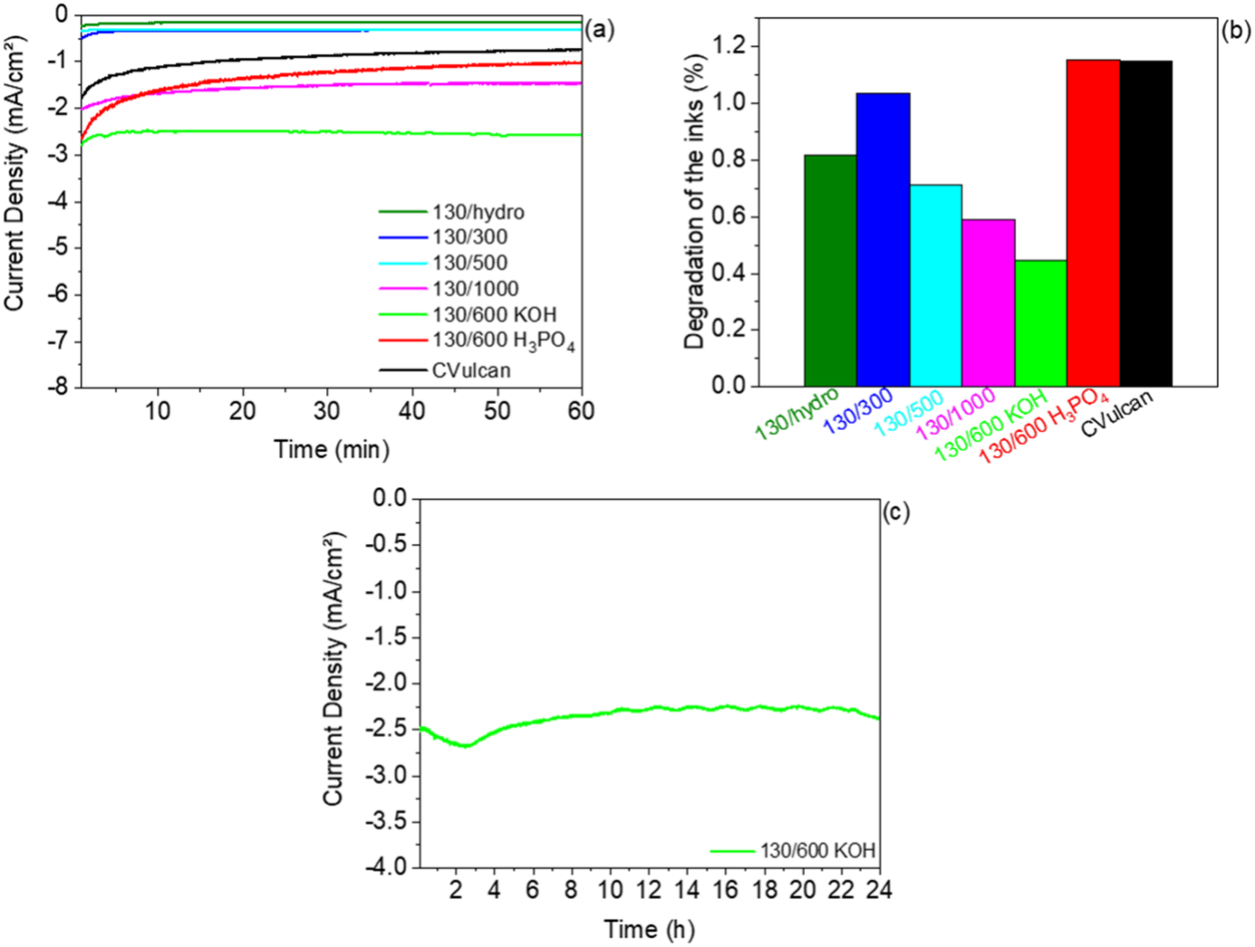
(a) CA stability studies for contaminated biomass catalysts and CVulcan during 1 h with a 0.1 M NaOH + 1 M Na_2_SO_4_ solution at −1400 mV vs Hg/HgO; (b) degradation percentage of each electrode, and (c) CA stability studies for 130/600 KOH during 24 h with a 0.1 M NaOH + 1 M Na_2_SO_4_ solution at −1400 mV vs Hg/HgO.

In conclusion, it can be stated that, in general terms, the materials studied exhibit significant activity, with the two chemically activated compounds (130/600 KOH and 130/600 H_3_PO_4_) and the material activated at 1000 °C (130/1000) showing activity levels surpassing that of the reference carbon Vulcan sample. This improvement can be attributed to several factors:

(1) The higher electrochemically active surface area of these materials, which is 46.40 cm^2^ for the KOH-activated sample and 9.45 cm^2^ for the H_3_PO_4_-activated sample, compared to 7.74 cm^2^ for the carbon Vulcan reference.
(2) The presence of structural defects, characterized through Raman spectroscopy. The *I*_D_/*I*_G_ values were 0.99 for 130/1000, 0.92 for 130/600 KOH, and 0.92 for 130/600 H_3_PO_4_, which are higher than those of the other samples, indicating more efficient electron transfer behavior. Additionally, the A_D_/A_G_ values were 1.49, 1.63, and 1.78, respectively. These results suggest that all three catalysts exhibit a high level of defects in their carbonaceous structures, which create additional active sites that enhance the HER.
(3) The confirmation of C = O functional groups through XPS, which may influence the interaction of the electrodes with the reactants.
(4) The presence of C = N groups identified by both XPS and FT-IR, which affect the electronic properties and catalytic activity.
(5) The detection of metals in the materials, confirmed by XRD and XPS, which may play a key role in electrocatalytic activity.

Among all the materials, the KOH-treated sample stands out as the most efficient, which can be justified not only by the analysis of its electrochemical surface area (ECSA), but also by the presence of K-related groups (such as K_2_ZnSiO_4_) identified by XRD, and the remarkable intensity of the C = O peak observed in the XPS analysis. These results suggest that the treatment with KOH significantly contributes to the enhancement of electrocatalytic activity in hydrogen production. Although the performance of Pt/C catalysts, which are commonly used for hydrogen production, is typically higher than that of these carbon-based materials derived from organic waste, better efficiencies to traditional carbon blacks have been achieved without the need of adding CRMs to the active phase of the catalyst.^78,79^ These better efficiencies, combined with the environmental and economic advantages, make these carbon materials from organic waste a promising alternative that could replace conventional carbon blacks in the near future. Therefore, the use of organic waste addresses pressing waste disposal concerns while offering a cost-effective solution for electrode production.

## 4 Conclusions

A sustainable and low-cost route has been developed for the fabrication of catalysts from biomass-derived carbon materials. The HTC process enabled the valorization of biomass into various functional materials (hydrochar, thermally activated, and thermally and chemically activated samples) demonstrating their potential for electrochemical hydrogen production. The CVulcan was used as a reference material to evaluate performance in hydrogen evolution reaction (HER). The results show that the catalyst chemically activated with KOH exhibits the best electrocatalytic activity for hydrogen production, with the LSV curve being nearly three times more pronounced than that of the reference carbon black. In addition, the 130/600 KOH sample shows an outstanding Tafel slope (147 mV/dec) and a low overpotential (−550 mV vs RHE), clearly surpassing the values obtained for CVulcan. Furthermore, a remarkable ECSA value of 46.40 cm^2^ is achieved, which is six times greater than that obtained for CVulcan. The impressive electrochemical performance of 130/600 KOH is corroborated by its strong results in CA and EIS techniques. For the thermally activated samples, catalytic performance improved with increasing temperature, yielding residual results up to 500 °C, where slight electrochemical activity is observed. The sample activated at 1000 °C demonstrates high electrocatalytic activity, surpassing both 130/600 H_3_PO_4_ and CVulcan, except in terms of ECSA, due to issues of heterogeneity arising from subjecting the catalyst to such high temperatures. Studies using various physicochemical characterization methods reveal that metals such as Si, Al, Fe, and Zn remain present in the catalysts in the form of chemical compounds. This study confirms that plant biomass waste containing metals can serve as an effective alternative to carbon black as electrocatalysts for hydrogen production, as carbon blacks are commonly used as support for catalysts in the HER and are manufactured from fossil foils. Moreover, this approach offers a sustainable and efficient solution for the valorization of such wastes.

## Supplementary Material



## Notes

Schematic diagram of the synthesis process from ; full XRD patterns with peak identification; Raman deconvolutions of all samples; XPS survey spectra; high-resolution XPS spectra of metals and elements in contaminated biomass catalysts (Si, Zn, Al, Fe, Ca, P); comparative LSV and chronoamperometry in different electrolytes; overpotential comparison at 5 mA cm^–2^; CV curves at various scan rates for selected electrodes in mixed alkaline electrolyte for the ECSA calculations (PDF)
