# Supplementary material for: Turning Polluted Biomass Waste into Sustainable Carbon-Based Catalysts for Hydrogen Production via Water Electrolysis
Source: Energy Fuels. 2025 Jul 23;39(31):15003–15. doi: 10.1021/acs.energyfuels.5c02282 (PMC12337815; doi:10.1021/acs.energyfuels.5c02282)
Supplement: Supplementary file 1 [file ef5c02282_si_001.pdf]

Supplementary Material

Turning Polluted Biomass Waste into Sustainable Carbon-Based Catalysts for Hydrogen Production via Water Electrolysis

Jorge Comendador, Javier Llanos, Álvaro Ramírez, Martín Muñoz-Morales, Ester López-Fernández*

Department of Chemical Engineering. Faculty of Chemical Sciences and Technologies. University of Castilla La Mancha. Campus Universitario s/n. 13071 Ciudad Real. Spain.

***Corresponding author**: ester.lfernandez@uclm.es

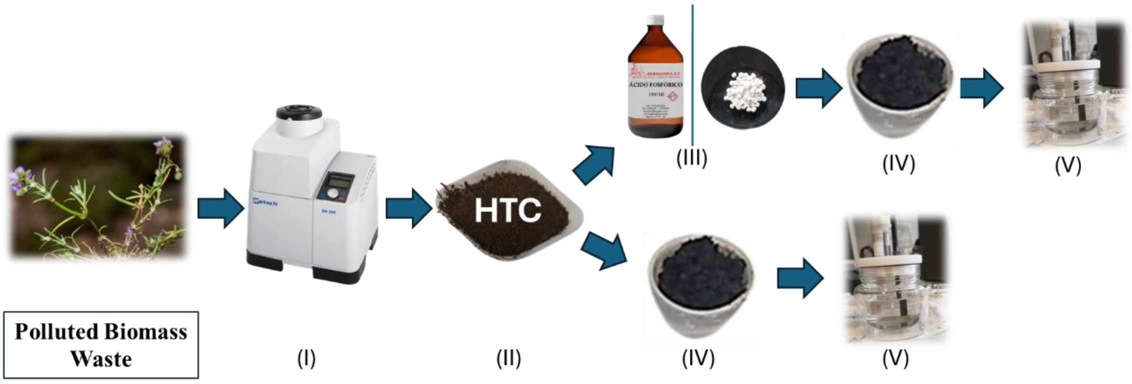

Figure S1. Schematic diagram of the synthesis of carbon material from *Spergularia rubra*.

All the XRD peaks:

- **130/hydro:**

SiO_2 (quartz): 24.4º, 50.2º, 68.0º

C_2CaO_4: 15.0º, 26.7º, 38.2º, 50.2º

Fe_3O_4: 18.5º, 30.0º, 43.3º

SiC: 35.8º, 38.2º, 43.3º, 50.2º

O_2: 30.0º

- **130/300:**

SiO_2: 15.0º, 26.7º, 29.8º, 36.7º, 39.5º, 42.4º, 45.8º, 50.1º, 55.2º, 60.0º, 68.3º, 81.3º

$Fe_3Al_2(SiO_4)_3$: 35.0º, 36.7º, 39.5º, 50.1º, 55.2º, 60.0º, 68.3º

Ca: 29.8º, 45.8º, 55.2º, 60.0º, 68.3º

$Al_{25}Mg_{37.5}Zn_{37.5}$: 36.7º, 50.1º

- **130/500:**

SiO_2: 20.8º, 26.7º, 36.6º, 39.4º, 40.2º, 42.4º, 45.8º, 50.1º, 54.8º, 59.9º, 68.1º, 74.4º, 75.5º, 79.9º, 90.8º, 94.9º

ZnO: 31.9º, 34.7º, 47.5º, 68.1º, 81.2º

$Fe_3Al_2(SiO_4)_3$: 34.7º, 36.6º, 50.1º, 59.9º, 68.1º, 74.4º, 75.5º

PbO: 31.9º, 34.7º, 42.4º, 45.8º, 54.9º, 74.4º, 75.5º, 79.9º

$Al_{12}Mg_{17}$: 20.8º, 26.7º, 31.9º, 36.6º, 50.1º, 54.8º, 74.4º, 81.2º

- **130/1000:**

SiO_2: 20.8º, 26.7º, 31.9º, 36.6º, 39.4º, 40.2º, 42.4º, 45.8º, 50.1º, 54.8º, 59.9º, 63.1º, 68.1º, 74.4º, 75.5º, 79.9º, 90.8º, 94.9º

Fe_2O_3: 35.8º, 55.0º, 63.1º, 75.8º

$CaSiO_3$: 35.8º, 45.9º, 63.1º, 77.7º

O_2: 26.1º, 39.6º, 42.6º, 63.1º

- **130/600 KOH:**

K_2ZnSiO_4 y $K_2Al_2O_4$: 20.0º, 32.9º, 38.7º, 47.7º, 58.7º, 68.9º, 78.4º, 96.8º

$Mg_2(SiO_4)$: 38.7º

- **130/600 H_3PO_4:**

SiO_2: 26.1º, 68.8º, 75.2º

$(Al_{0.47}Si_{0.13}P_{0.40})O_2$: 24.2º

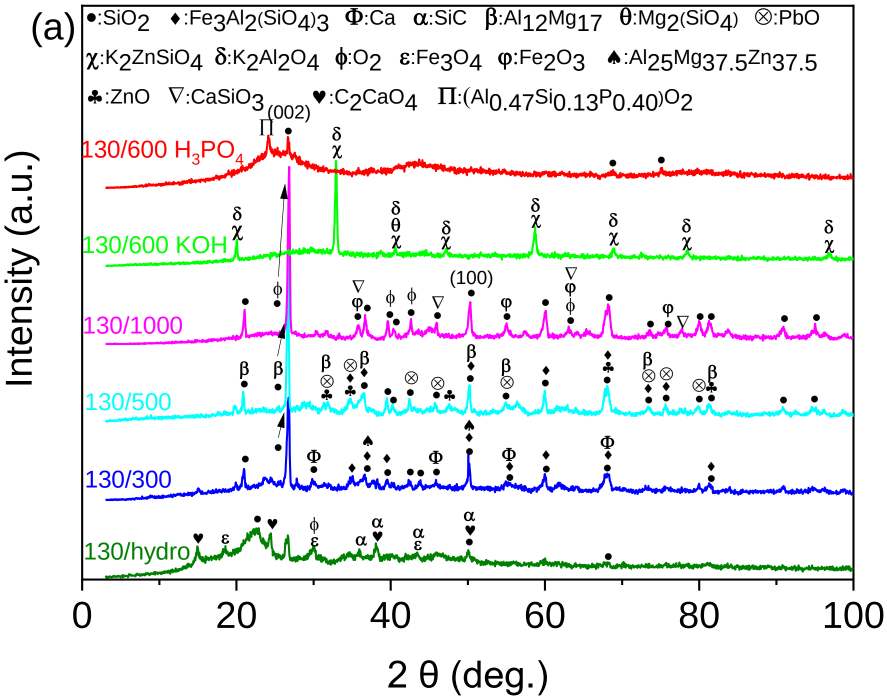

Figure S2. XRD patterns (all peaks).

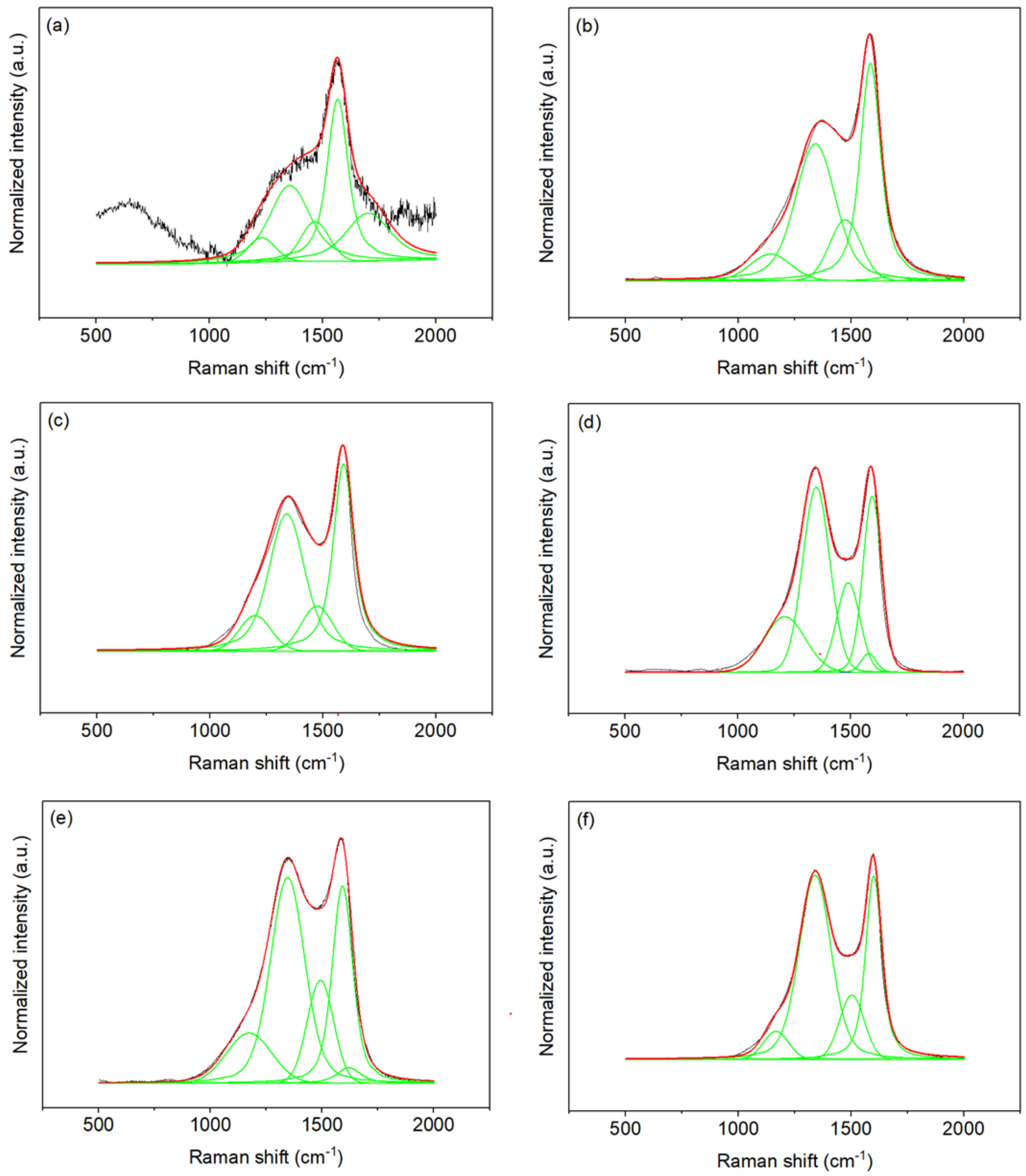

Figure S3. Raman deconvolutions for (a) 130/hydro, (b) 130/300, (c) 130/500, (d) 130/1000, (e) 130/600 KOH and (f) 130/600 H_3PO_4.

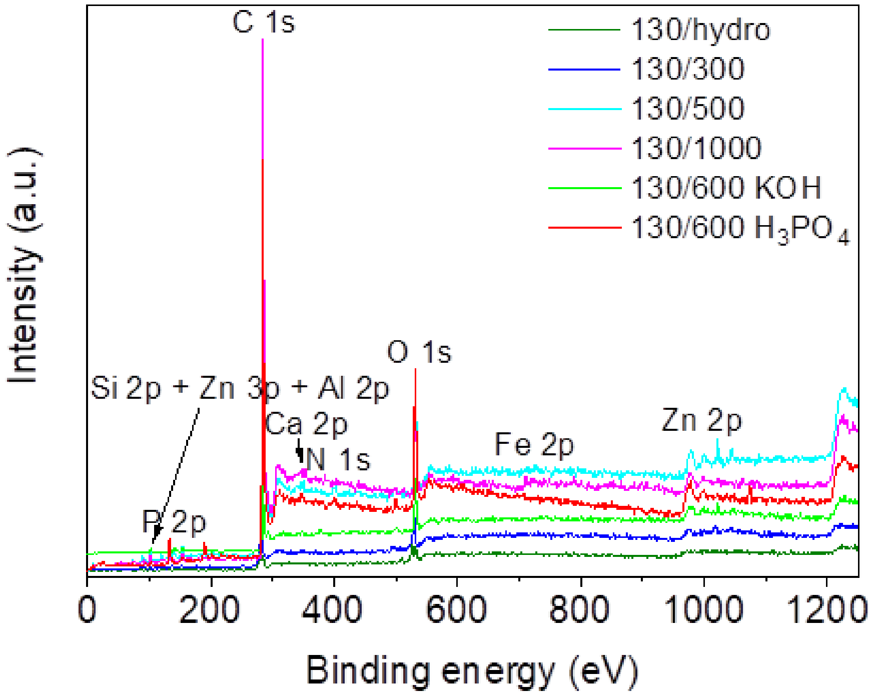

Figure S4. XPS survey spectra.

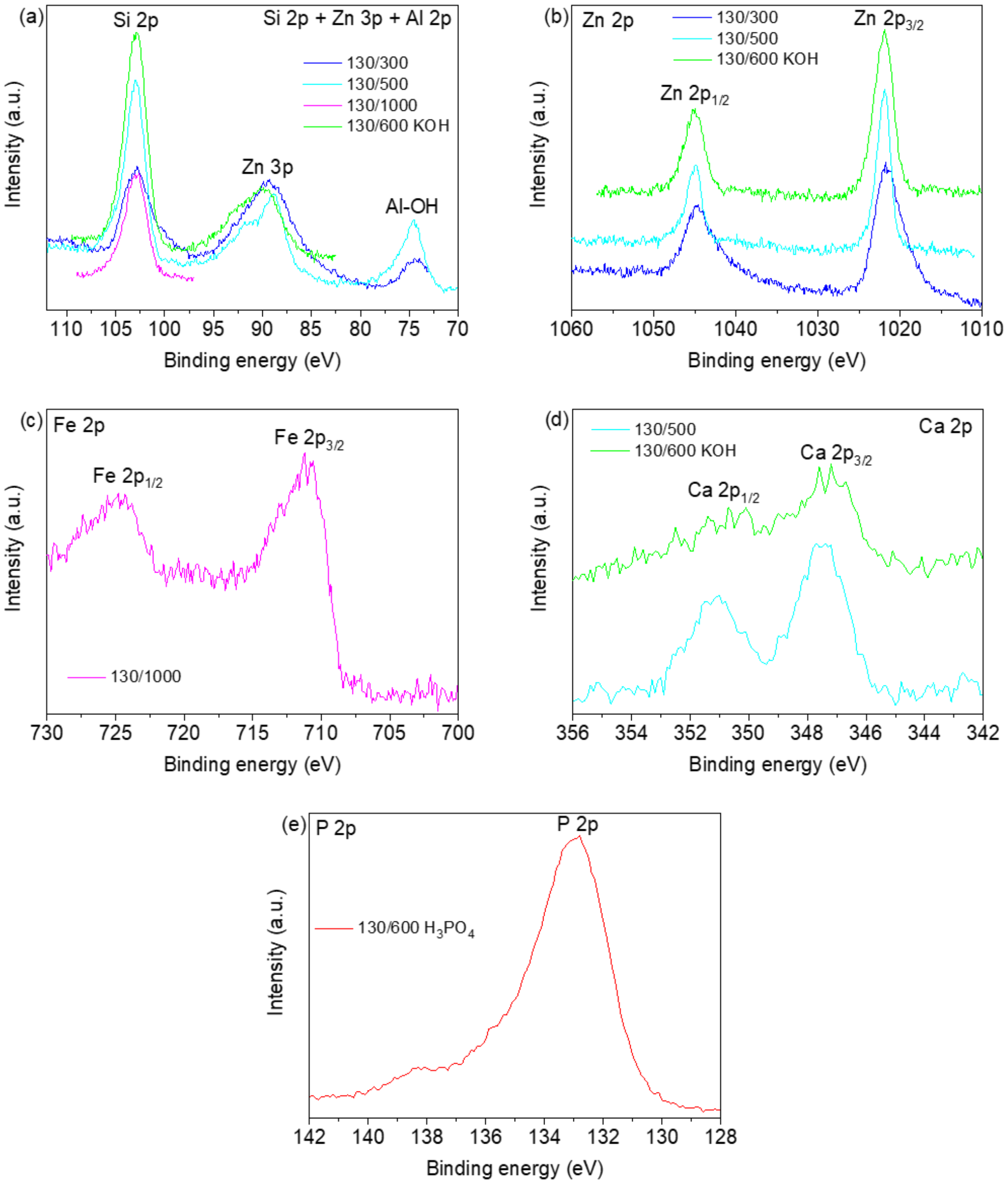

Figure S5. XPS survey spectra of contaminated biomass catalysts: (a-e) high-resolution spectra of (a) Si 2p + Zn 3p + Al 2p, (b) Zn 2p, (c) Fe 2p, (d) Ca 2p and (e) P 2p, respectively.

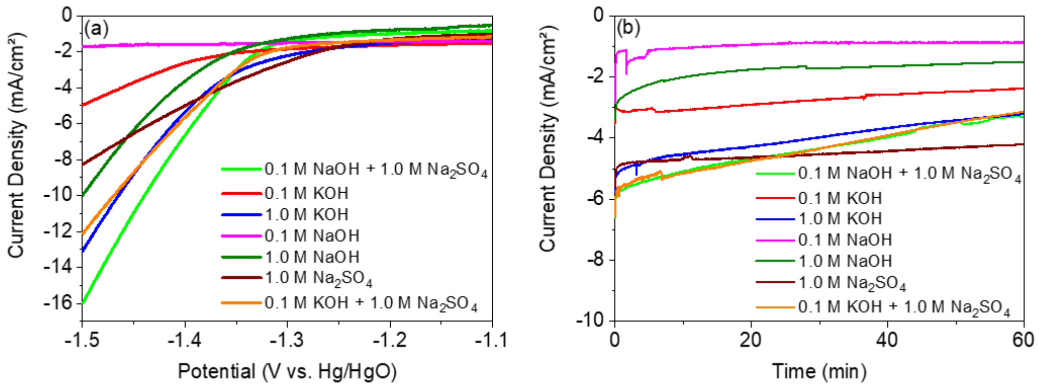

Figure S6. Comparative electrolytes for (a) LSV and (b) CA.

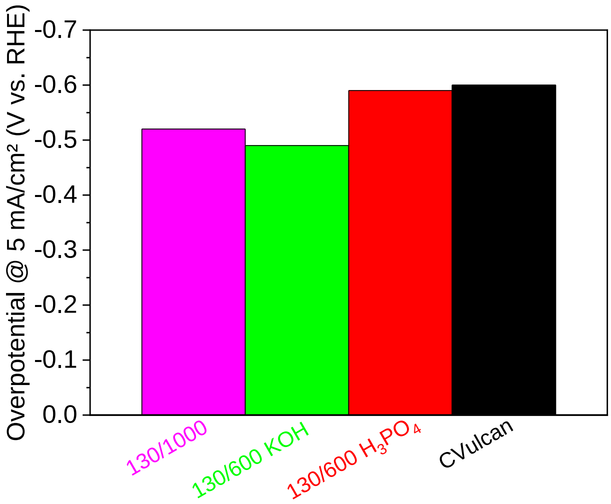

Figure S7. Comparison of overpotentials at a current density of 5 mA/cm².

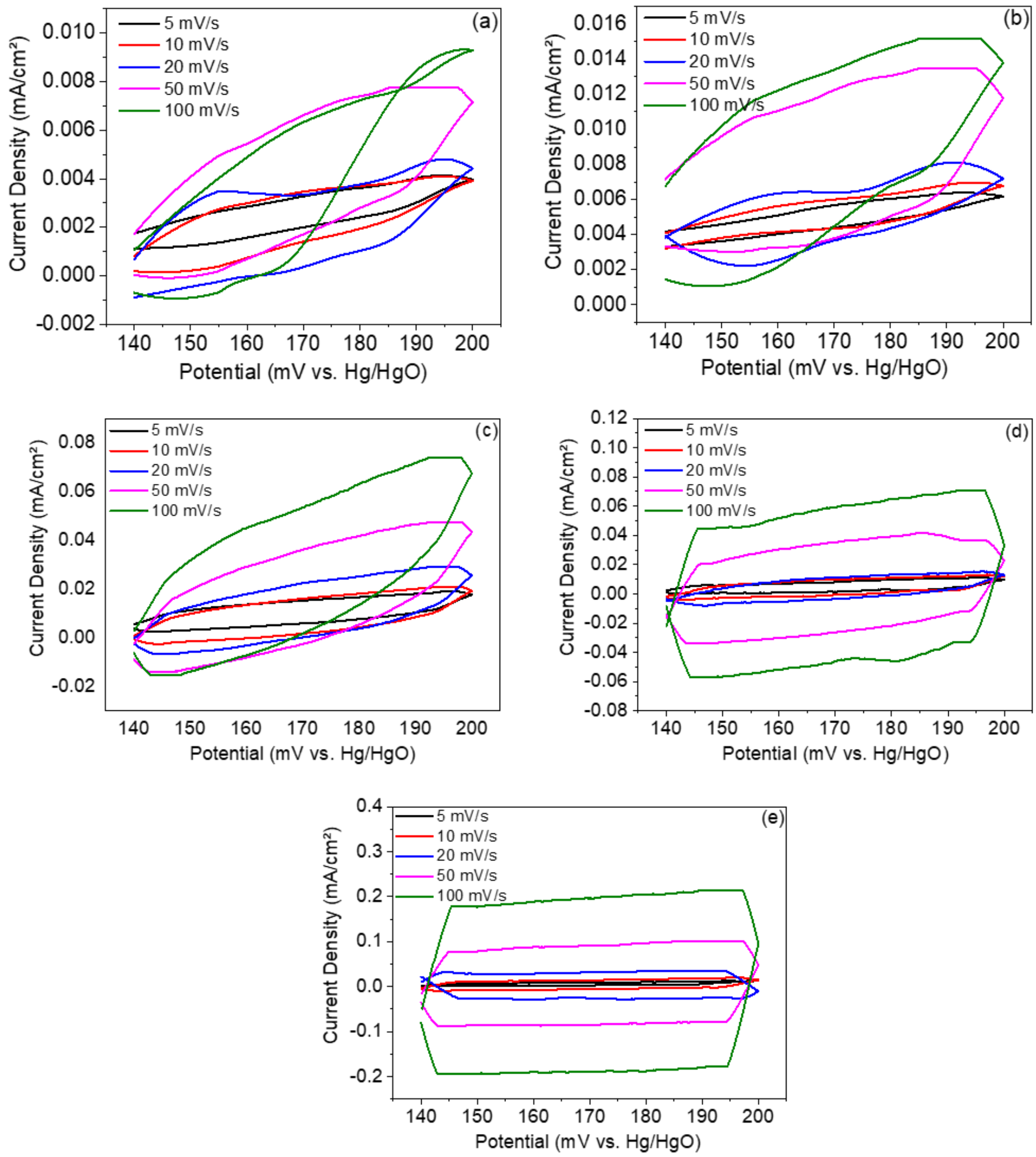

Figure S8. CV plots recorded at scan rates ranging from 5 to 100 mV/s for the (a) 130/hydro, (b) 130/300, (c) 130/500, (d) 130/1000 and (e) 130/600 H_3PO_4 electrodes samples with a 0.1 M NaOH + 1 M Na_2SO_4 solution.
